# Relationship Between External Ear Severity and Temporal Bone Malformation in Microtia: A Computed Tomography Study

**DOI:** 10.3390/jcm15187031

**Published:** 2026-09-10

**Authors:** Ekrem Solmaz, Gokce Yildiran, Zeliha Fazliogullari, Zekeriya Tosun

**Affiliations:** 1 Department of Anatomy, Faculty of Medicine, Selcuk University, Selcuklu, Konya 42131, Turkey; gokceyildiran@gmail.com (G.Y.); z_topal@yahoo.com (Z.F.); 2 Department of Plastic, Reconstructive and Aesthetic Surgery, Faculty of Medicine, Selcuk University, Selcuklu, Konya 42131, Turkey; drtosunz@gmail.com

**Keywords:** microtia, temporal bone, computed tomography, diagnostic imaging, Jahrsdoerfer score

## Abstract

**Background/Objectives**: Microtia is frequently associated with temporal bone malformations, but the extent to which external ear phenotype reflects underlying CT-defined anatomy remains uncertain. This study aimed to compare the relationships of Marx severity grading and Nagata morphological classification with temporal bone anatomy in patients with microtia. **Methods**: This retrospective study included 26 patients with 27 affected ears who had both preoperative external ear photographs and temporal bone computed tomography (CT) examinations. Each affected ear was classified according to the Marx and Nagata systems, and temporal bone anatomy was assessed using the modified Jahrsdoerfer CT scoring system. Associations were evaluated using Spearman rank correlation and permutation-based Jonckheere–Terpstra analysis, with an additional patient-level sensitivity analysis. **Results**: Considerable variation in Jahrsdoerfer scores was observed within external ear phenotypic categories, with substantial overlap between Marx Grades II and III and lower scores concentrated in Grade IV. The inverse association between Marx grade and Jahrsdoerfer score did not reach conventional statistical significance (ρ = −0.380, *p* = 0.051), whereas the Jonckheere–Terpstra test identified an overall decreasing trend (permutation *p* = 0.025). This trend no longer reached statistical significance in the patient-level sensitivity analysis. Nagata classification did not significantly discriminate CT-based radiological severity. **Conclusions**: Greater external auricular severity may be associated with less favorable temporal bone anatomy, but the relationship was not uniform across successive Marx grades. Marx and Nagata classifications provide complementary phenotypic information, but neither consistently reflected the extent of underlying temporal bone malformation. These preliminary findings emphasize phenotype–CT variability and support individualized CT-based anatomical assessment when imaging is clinically indicated.

## 1. Introduction

Microtia is a congenital anomaly of the auricle that is frequently associated with additional structural abnormalities and represents the most common developmental defect of the external ear. Its estimated incidence is approximately 1 in 10,000 live births [1], with bilateral involvement reported in about 10% of cases [2,3]. Microtia frequently coexists with congenital aural atresia, a condition characterized by an underdeveloped external auditory canal and malformed middle ear ossicles, and has been reported to occur in approximately 1 in 10,000 to 20,000 live births [4,5]. This anomaly may affect not only the external auditory canal but also the auricle, middle ear components, and, in some cases, the inner ear [5]. The severity of microtia varies widely, ranging from a slightly reduced yet morphologically normal auricle to rudimentary soft tissue remnants or near-complete absence of the auricle. External auditory canal anomalies associated with congenital aural atresia may range from partial stenosis to complete atresia [6]. Middle ear abnormalities may include stapes malformation, absence of the oval and/or round windows, aberrant facial nerve course, inadequate middle ear pneumatization, and fusion of the malleus and incus [7,8].

Several classification systems have been developed to characterize the external auricular phenotype, but they describe different aspects of microtia. The Marx-based classification primarily grades the developmental severity of auricular deficiency, whereas the Nagata classification categorizes the morphology of the auricular remnant into concha, small concha, lobule, and anotia types within a framework originally developed for reconstructive planning [9,10,11,12]. These systems should therefore be regarded as complementary rather than interchangeable descriptions of the external ear phenotype. A recent scoping review of contemporary reconstructive literature confirmed the predominant use of the Nagata classification while documenting the continued, although less frequent, use of other classification systems, including Marx-based grading [13].

High-resolution computed tomography (CT) provides detailed assessment of surgically relevant temporal bone anatomy and plays an important role in the preoperative evaluation of patients with microtia and external auditory canal abnormalities [14,15,16]. The Jahrsdoerfer framework provides a 10-point grading approach for assessing anatomical suitability for congenital aural atresia surgery [16,17,18]. Previous studies have demonstrated a broad relationship between auricular morphology and middle-ear or temporal-bone development, with less developed auricular phenotypes generally associated with less favorable radiological anatomy [2,19,20,21]. However, this relationship is not uniform across individual anatomical structures or phenotypic categories.

Although the general association between external ear development and underlying temporal bone anatomy has been established, the comparative value of conceptually distinct external-ear classification frameworks remains less well characterized. Comparing a severity-oriented system such as Marx grading with a morphology- and reconstruction-oriented system such as the Nagata classification within the same cohort may clarify whether these phenotypic frameworks differ in their relationship with CT-based temporal bone anatomy. Such information may allow external auricular morphology to provide preliminary contextual information during clinical counselling, but it should be recognized that external appearance cannot substitute for individualized CT assessment.

Accordingly, the present study aimed to compare the relationships of Marx-based severity grading and Nagata morphological classification with CT-based temporal bone anatomy in patients with microtia, and to evaluate the extent of radiological variability within phenotypically similar external-ear categories.

## 2. Materials and Methods

### 2.1. Study Population

**Figure 1:**
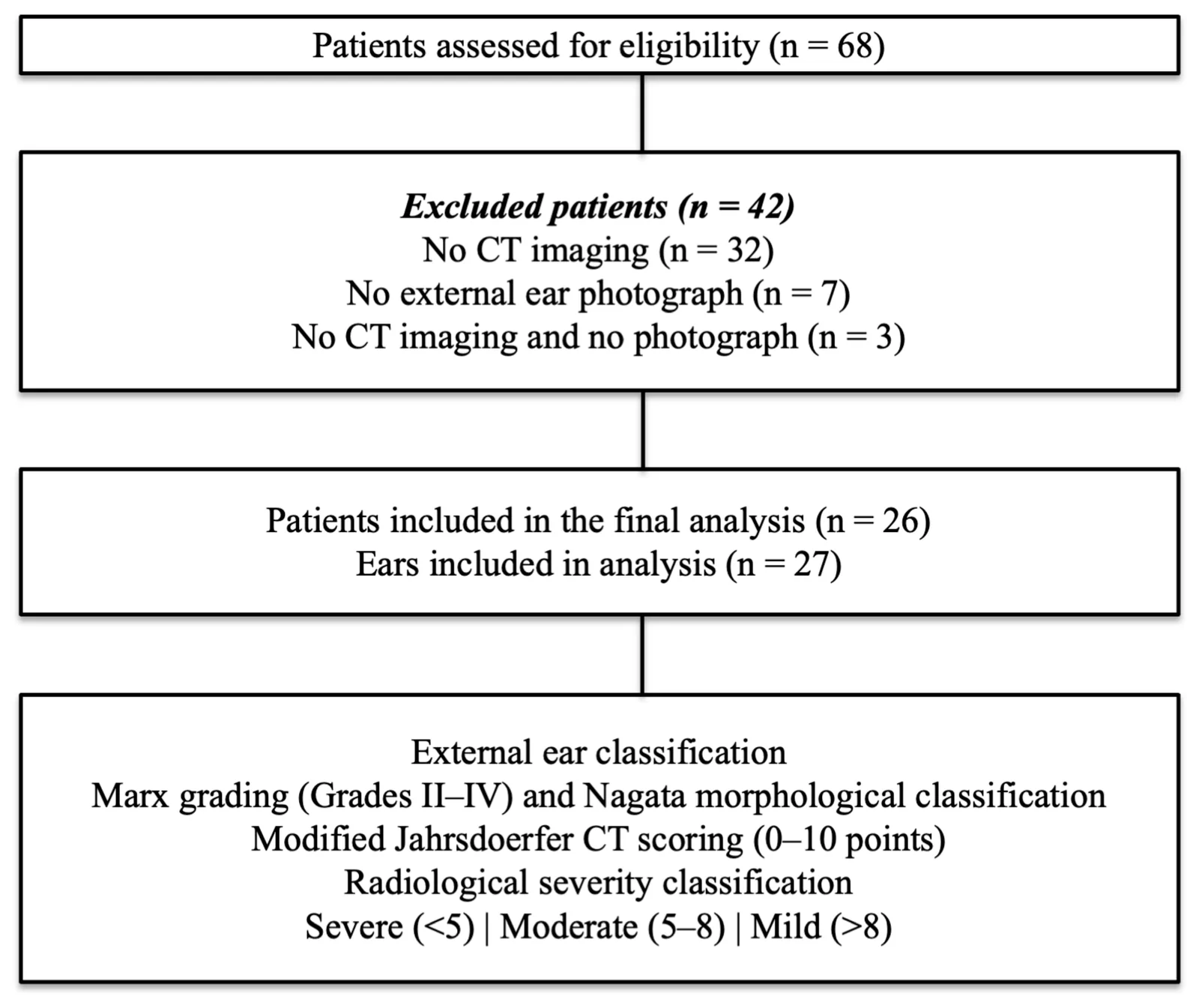
Flowchart of study population selection and subsequent phenotypic and radiological assessment.

This retrospective study evaluated patients with microtia who obtained both preoperative external ear photographs and temporal bone CT examinations between 2010 and 2023. A total of 68 patients were assessed for eligibility. Patients without available CT imaging (*n* = 32), without an external ear photograph (*n* = 7), or without either CT imaging or an external ear photograph (*n* = 3) were excluded. Consequently, 26 patients with 27 affected ears were included in the final analysis (Figure 1).

This retrospective study was approved by the local institutional ethics committee, with exemption for the requirement for written informed consent; specifically, the Selcuk University Faculty of Medicine Local Ethics Committee approved the study (decision No. 2024/398). Preoperative external ear photographs retrieved from the patients’ records were used for phenotypic classification of microtia, whereas the corresponding temporal bone CT examinations were used for radiological assessment.

### 2.2. External Ear Classification

**Figure 2:**
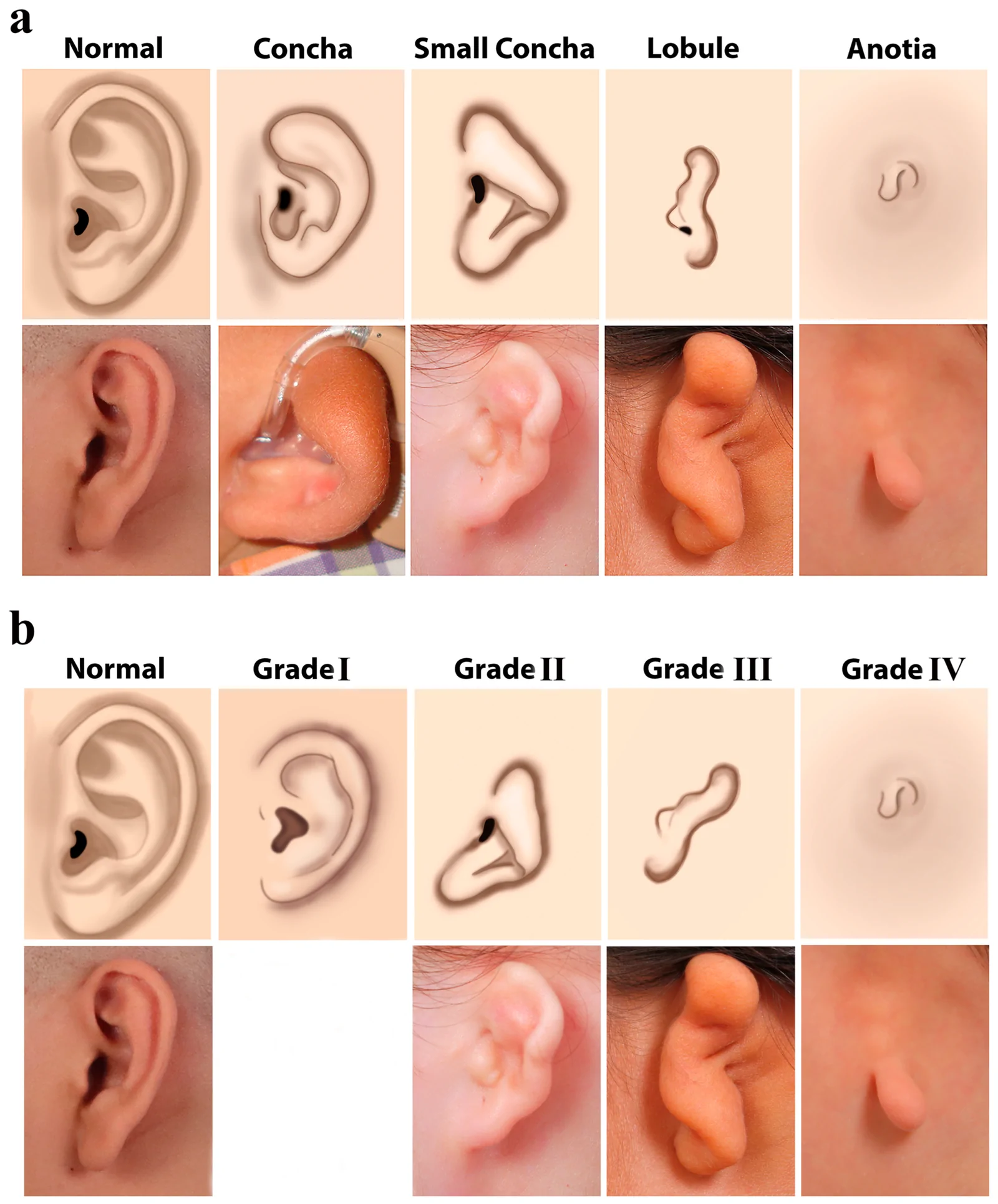
Schematic illustrations and representative patient photographs of microtia according to the (**a**) Nagata morphological classification and (**b**) Marx severity classification. No patient with Marx Grade I microtia was present in the study cohort; therefore, no corresponding clinical photograph is shown for Grade I.

**Figure 3:**
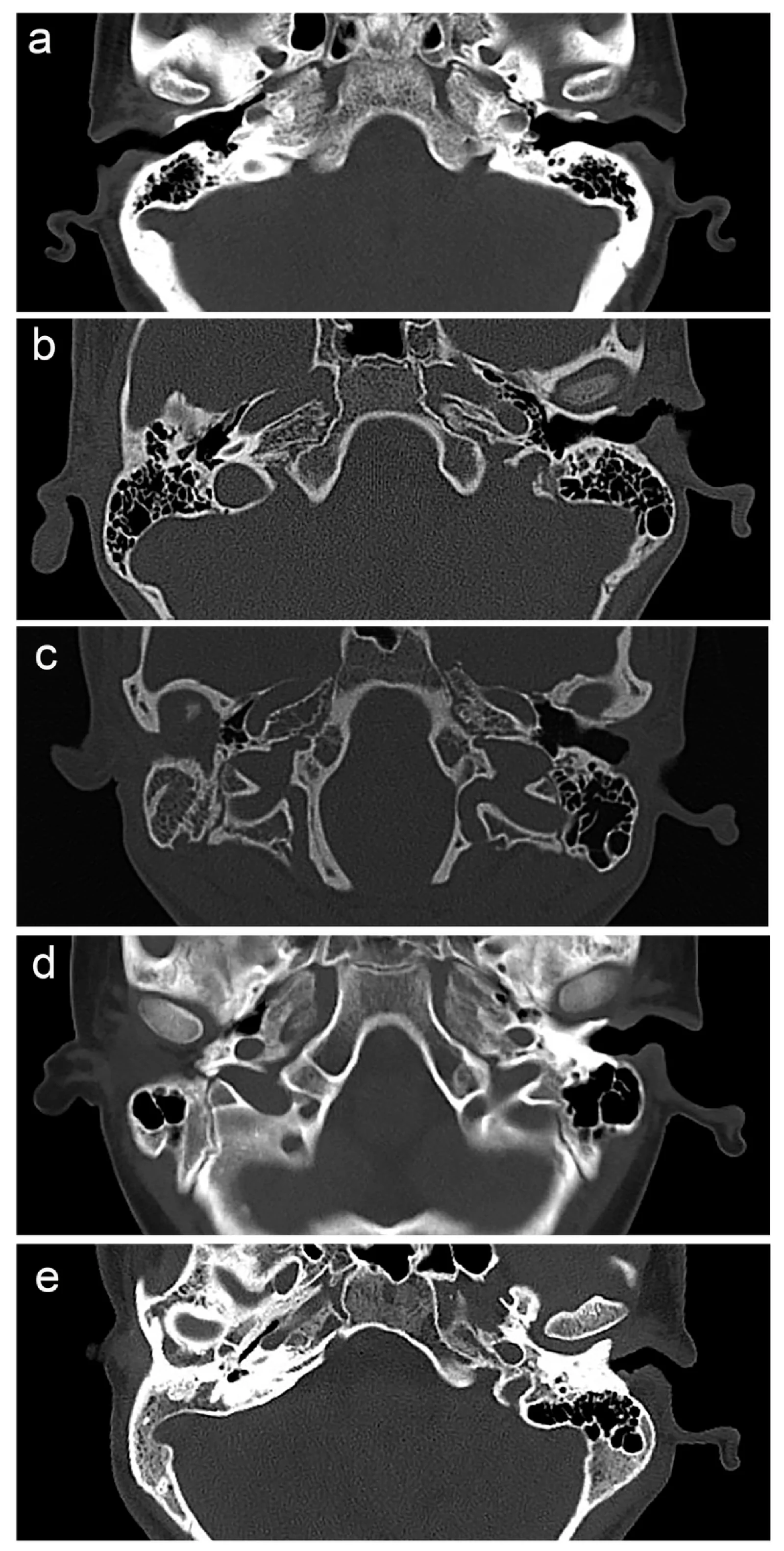
Representative axial computed tomography images illustrating the CT appearance of different auricular phenotypes in one healthy individual and patients with right-sided microtia associated with external auditory canal atresia, classified according to the Nagata system. (**a**) Normal auricle with a patent external auditory canal. (**b**) Concha-type microtia in a 6-year-old male patient. (**c**) Small concha-type microtia in a 15-year-old female patient. (**d**) Lobule-type microtia in an 11-year-old male patient. (**e**) Anotia in a 17-year-old male patient.

Preoperative external ear photographs were retrospectively evaluated by the plastic surgeon responsible for the patients’ clinical care using both the Marx-based grading system and the Nagata classification. The Marx-based system was used to grade the severity of auricular developmental deficiency as Grades I–IV [9,10,11]. In the present cohort, affected ears were classified as Grades II–IV, as no Grade I cases were identified. The Nagata system was used to classify auricular morphology as concha type, small concha type, lobule type, or anotia [12]. Each affected ear was assigned both a Marx grade and a Nagata morphological subtype, allowing the relationships of the two phenotypic classification approaches with CT-defined temporal bone anatomy to be evaluated within the same cohort (Figure 2). Representative CT appearances of the different Nagata morphological phenotypes are shown in Figure 3.

### 2.3. Radiological Assessment of Temporal Bone Anatomy

Temporal bone CT images of each affected ear were evaluated using the modified Jahrsdoerfer CT scoring system [2,17]. In the modification described by Ishimoto et al. [2], the “appearance of external ear” parameter of the original Jahrsdoerfer system was replaced by “external ear canal present”, thereby allowing external auricular morphology to be evaluated separately using the Marx classification. The modified system has a maximum score of 10 points: the presence of the stapes is assigned 2 points, whereas the oval window, round window, middle ear space, facial nerve, malleus–incus complex, mastoid pneumatization, incus–stapes connection, and external auditory canal are each assigned 1 point. Higher total scores indicate more favorable temporal bone anatomy. For the external auditory canal component, complete canal atresia was scored as absent (0 points), whereas a stenotic but present canal was scored as present (1 point).

### 2.4. CT Acquisition

Scanning was performed using a 256-slice multi-detector CT scanner (SOMATOM Definition Flash, Siemens Healthcare, Erlangen, Germany). Imaging parameters were as follows: 120 kV; 160 mA; rotation time, 0.5 s; collimation, 64 × 0.625 mm; and field of view (FOV), 220 mm. Images of the temporal bone region were retrospectively evaluated on a workstation (syngo.via [VB30A], Siemens Healthineers, Erlangen, Germany).

### 2.5. Statistical Analysis

Statistical analyses were performed using R software (version 4.5.2; R Foundation for Statistical Computing, Vienna, Austria). Continuous variables were expressed as the median and interquartile range (IQR), whereas categorical variables were presented as numbers and percentages. Each affected ear was considered the unit of analysis in the primary ear-level analyses. The association between external ear anomaly severity and radiological score was evaluated using Spearman’s rank correlation analysis. Because increasing Marx grade represents greater external ear severity, whereas lower Jahrsdoerfer scores represent less favorable temporal bone anatomy, an ordered decrease in Jahrsdoerfer scores across increasing Marx grades was additionally evaluated using the Jonckheere–Terpstra trend test. Permutation-based *p*-values were calculated using 100,000 permutations with a prespecified decreasing alternative and a fixed random seed of 20260825. Associations between categorical variables were evaluated using the Fisher–Freeman–Halton exact test for contingency tables larger than 2 × 2. Differences in radiological scores between two independent groups were analyzed using the Wilcoxon rank-sum test, whereas comparisons across multiple groups were performed using the Kruskal–Wallis test. Because one patient had bilateral microtia and therefore contributed two affected ears to the primary ear-level analysis, a patient-level sensitivity analysis was additionally performed to assess the potential effect of within-patient dependence. For this analysis, one affected ear from the bilateral patient was excluded, resulting in 26 independent patient-level observations, and the Spearman rank correlation and permutation-based Jonckheere–Terpstra analyses were repeated using the same test direction, number of permutations, and random seed. The Spearman correlation and permutation-based Jonckheere–Terpstra analyses were independently recalculated and verified using Python (version 3.13) with the SciPy (version 1.17) and NumPy (version 2.4) libraries. For nondirectional statistical tests, a two-sided *p*-value < 0.05 was considered statistically significant; the Jonckheere–Terpstra test was evaluated using the prespecified decreasing alternative.

### 2.6. Artificial Intelligence Use

Artificial intelligence-assisted tools, including OpenAI ChatGPT (GPT-5.5, OpenAI, San Francisco, CA, USA), DeepL Write (https://www.deepl.com/write, accessed on 1 June 2026), and Grammarly (https://www.grammarly.com/, accessed on 1 June 2026), were used during manuscript preparation and revision to assist with language editing, improve clarity, and refine the presentation of the text. The graphical abstract was generated using AI-assisted visualization tools (OpenAI ChatGPT/DALL·E; https://openai.com/, accessed on 18 July 2026) and subsequently critically reviewed, revised, and approved by the authors, who assume full responsibility for its scientific accuracy and content. AI-assisted tools were not used for patient selection, external ear classification, CT interpretation, Jahrsdoerfer scoring, or generation of the study data. All scientific interpretations and conclusions were reviewed and verified by the authors, who take full responsibility for the final manuscript.

## 3. Results

A total of 26 patients with 27 affected ears were included in the analysis. The median age was 14 years (interquartile range [IQR]: 11–18 years). Nineteen patients (73.1%) were male and seven (26.9%) were female. Bilateral microtia was identified in 1 patient (3.8%), resulting in 2 affected ears in the same individual, whereas the remaining 25 patients (96.2%) had unilateral involvement. Among the affected ears, 15 (55.6%) were right-sided and 12 (44.4%) were left-sided (Table 1). Complete external auditory canal atresia was present in 24 of 27 affected ears (88.9%), whereas three ears (11.1%) had a stenotic but present external auditory canal; no affected ear had a normally patent external auditory canal.

According to the Marx classification, external ear anomalies were categorized as Grade II in 6 ears (22.2%), Grade III in 16 ears (59.3%), and Grade IV in 5 ears (18.5%). No Grade I cases were identified in the study cohort. Based on the Nagata classification, lobule-type microtia was the most frequent morphological subtype, observed in 16 ears (59.3%), followed by anotia in 5 ears (18.5%), while concha-type and small concha-type microtia were each identified in 3 ears (11.1%).

**Figure 4:**
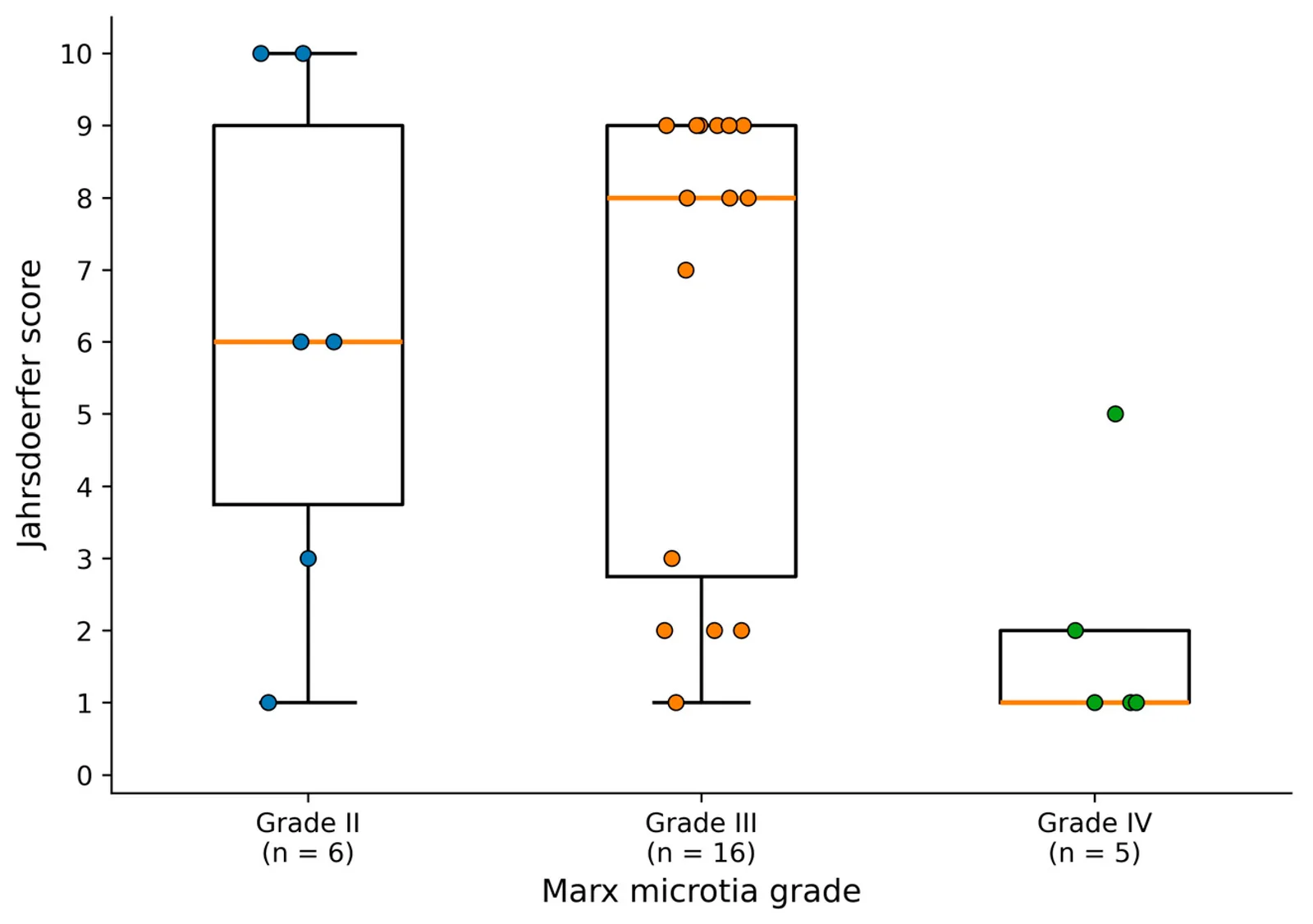
Distribution of Jahrsdoerfer scores across Marx microtia grades. Box plots show the median and interquartile range, with individual affected ears displayed as jittered points. Orange horizontal lines indicate the median, and point colors denote Marx grade (blue, Grade II; orange, Grade III; green, Grade IV). Lower Jahrsdoerfer scores indicate less favorable temporal bone anatomy.

Jahrsdoerfer scores ranged from 1 to 10, with lower scores indicating less favorable temporal bone anatomy and a greater burden of associated structural abnormalities. The median scores were 6.0 (3.75–9.0) for Marx Grade II, 8.0 (2.75–9.0) for Grade III, and 1.0 (1.0–2.0) for Grade IV (Table 2). Individual score distributions showed substantial overlap between Grades II and III, and the median score was higher in Grade III than in Grade II. In contrast, Grade IV ears were concentrated at the lower end of the Jahrsdoerfer score range (Figure 4).

Exploratory examination of individual Jahrsdoerfer components showed additional structural heterogeneity across Marx grades. Stapes, malleus–incus complex, and incudostapedial connection were absent in all Grade IV ears, whereas the facial nerve criterion remained relatively preserved across grades. Several individual components were present as frequently or more frequently in Grade III than in Grade II ears, consistent with the substantial overlap observed in total Jahrsdoerfer scores. Given the small subgroup sizes, these component-level observations were interpreted descriptively and no additional inferential comparisons were performed.

Based on the predefined Jahrsdoerfer score thresholds, 11 ears (40.7%) were classified as severe (<5), 7 (25.9%) as moderate (5–8), and 9 (33.3%) as mild (>8). Severe radiological involvement was observed in 2 of 6 Grade II ears (33.3%), 5 of 16 Grade III ears (31.3%), and 4 of 5 Grade IV ears (80.0%) (Table 3). However, the association between Marx grade and radiological severity category was not statistically significant (Fisher–Freeman–Halton exact test, *p* = 0.384).

When evaluated according to Nagata morphology, median Jahrsdoerfer scores were 6.0 (4.5–8.0) in the concha subtype, 6.0 (3.5–8.0) in the small concha subtype, 8.0 (2.75–9.0) in the lobule subtype, and 1.0 (1.0–2.0) in anotia. Severe radiological involvement was observed in 1 of 3 concha-type ears (33.3%), 1 of 3 small concha-type ears (33.3%), 5 of 16 lobule-type ears (31.3%), and 4 of 5 ears with anotia (80.0%). The association between Nagata subtype and radiological severity category was not statistically significant (Fisher–Freeman–Halton exact test, *p* = 0.590). Likewise, differences in Jahrsdoerfer scores among Nagata subtypes did not reach statistical significance (Kruskal–Wallis H = 7.07, *p* = 0.070).

Spearman rank correlation analysis showed a moderate inverse association between Marx grade and Jahrsdoerfer score (ρ = −0.380); however, this association did not reach conventional statistical significance (*p* = 0.051). The prespecified Jonckheere–Terpstra analysis identified an overall tendency toward lower ranked Jahrsdoerfer scores across increasing Marx grades (JT = 62.5, permutation *p* = 0.025). However, the group medians did not show a stepwise decrease across Marx grades, and substantial overlap was observed between Grades II and III (Figure 4). Therefore, the significant directional trend should be interpreted cautiously and does not indicate a uniform decrease in Jahrsdoerfer score between successive Marx grades. Because one patient contributed two affected ears, a patient-level sensitivity analysis was performed after excluding one ear from this bilateral case. In the resulting 26 independent patient-level observations, the inverse Spearman association was attenuated (ρ = −0.314, *p* = 0.119), and the decreasing trend identified by the Jonckheere–Terpstra test no longer reached statistical significance (JT = 61.5, permutation *p* = 0.060). These sensitivity findings indicate that the observed relationship should be regarded as preliminary and interpreted in the context of the small sample size.

## 4. Discussion

Microtia is a congenital developmental anomaly of the external ear that is frequently associated with variable temporal bone malformations, most commonly presenting unilaterally with male predominance and right-sided involvement [2,4]. In the present study, a similar epidemiological pattern was observed, with a male-to-female ratio of approximately 2.7:1 and right-sided involvement in 55.6% of affected ears. Bilateral involvement was identified in only one patient (3.8%), a proportion lower than that reported in larger clinical and imaging-based series [2,3,22]; however, this finding should be interpreted cautiously given the small sample size and single-center design.

The principal finding of the present study was an overall tendency toward less favorable CT-based temporal bone anatomy with greater Marx-defined external ear severity, although this relationship was not uniform across grades. Spearman rank correlation showed a moderate inverse association that did not reach conventional statistical significance (ρ = −0.380, *p* = 0.051), whereas the prespecified Jonckheere–Terpstra analysis identified a significant directional trend toward lower ranked Jahrsdoerfer scores with increasing Marx grade (permutation *p* = 0.025). Importantly, however, the median Jahrsdoerfer score was higher in Grade III than in Grade II, and substantial overlap was observed between these two groups. Moreover, the categorical association between Marx grade and radiological severity was not statistically significant (*p* = 0.384), and the directional trend no longer reached statistical significance in the patient-level sensitivity analysis (permutation *p* = 0.060). Taken together, these findings suggest a possible association between greater external auricular severity and less favorable temporal bone anatomy, but do not support a uniform stepwise relationship across successive Marx grades.

Previous CT-based studies have already demonstrated associations between external ear phenotype and temporal bone development in patients with microtia [2,20,21]. The present study therefore does not seek to establish this general association as a novel finding. Rather, its contribution lies in evaluating two conceptually different phenotypic classification approaches within the same cohort: the severity-oriented Marx grading system and the morphology-oriented Nagata classification. The considerable variation in Jahrsdoerfer scores observed within phenotypically similar categories, particularly the overlap between Marx Grades II and III, further indicates that external auricular phenotype does not consistently correspond to the extent of underlying temporal bone malformation. This comparison provides a more nuanced assessment of the relationship between external ear phenotype and CT-defined temporal bone anatomy than would be obtained from external severity grading alone.

The association between external auricular phenotype and temporal bone abnormalities is biologically plausible given the closely related embryological development of the external and middle ears. The auricle, external auditory canal, and major middle ear structures develop through coordinated processes involving the first and second pharyngeal arches [4,11,23,24]. This developmental relationship may contribute to the coexistence of external ear and temporal bone abnormalities; however, the substantial radiological variability observed within phenotypically similar categories in the present study indicates that external auricular severity does not uniformly reflect the extent of underlying temporal bone malformation.

Previous CT-based studies have similarly reported an association between external ear development and underlying middle ear anatomy [2,20,25]. Earlier work suggested that more severe microtia is generally associated with less favorable middle ear development [20]. In the study by Ishimoto et al. [2], total CT-based temporal bone scores were inversely correlated with Marx grade; however, significant differences were not observed between all adjacent severity groups, including Grades II and III. This finding is relevant to the present cohort, in which substantial overlap was also observed between Grades II and III and the Grade III median Jahrsdoerfer score was higher than that of Grade II. Thus, both previous and present findings suggest that an overall association between external auricular severity and temporal bone development should not be interpreted as a uniform stepwise relationship across successive external ear grades. Previous analyses have also indicated that this relationship may differ among individual temporal bone structures, with aeration-related parameters showing a stronger association with external ear severity than some ossicular and window-related components [2].

Previous CT-based studies have also demonstrated that developmental associations may differ among individual temporal bone structures [26,27]. Whereas some studies have specifically focused on surgically relevant structures such as the facial nerve [26,28], the present study used the modified Jahrsdoerfer score as a composite measure of temporal bone anatomy [2,17]. This approach permits multiple anatomical components to be summarized within a single radiological score. The exploratory component-level assessment further suggested that the phenotype–CT discordance was not confined to the composite score because individual temporal bone structures did not vary uniformly across successive Marx grades. Given the small subgroup sizes, however, these component-level observations should be interpreted descriptively rather than as evidence of structure-specific associations. Accordingly, the present findings primarily reflect differences in overall CT-based anatomical configuration rather than definitive structure-specific developmental associations.

The distribution of external ear severity in the present study was broadly comparable with prior clinical series in which Grade III microtia has frequently been reported as the predominant phenotype [10]. Similarly, Grade III represented the most common category in the present cohort. Despite this predominance, considerable radiological heterogeneity was observed: 11 of 27 affected ears (40.7%) were classified as severe, 7 (25.9%) as moderate, and 9 (33.3%) as mild according to the predefined Jahrsdoerfer score categories. Notably, severe radiological involvement was present across Marx Grades II–IV rather than being confined to the highest external ear severity grade, further illustrating the variability of temporal bone anatomy within external phenotypic categories.

Nagata-based classification provided complementary morphological characterization but did not significantly discriminate CT-based radiological severity in the present cohort. Although anotia had the lowest median Jahrsdoerfer score and the highest proportion of severe radiological involvement, neither the categorical association with radiological severity nor the differences in Jahrsdoerfer scores among Nagata subtypes reached statistical significance. This finding should be considered in light of the conceptual differences between the two external ear classification systems. The Marx system primarily grades the severity of auricular developmental deficiency, whereas the Nagata classification characterizes morphological subtypes with particular relevance to reconstructive planning [9,12,13]. Accordingly, the two systems should be regarded as complementary rather than interchangeable. In the present cohort, Marx grading showed an overall directional relationship with Jahrsdoerfer scores, whereas Nagata classification primarily provided morphological characterization; however, neither external classification consistently reflected the underlying CT-based temporal bone anatomy.

From a clinical perspective, the substantial variation in Jahrsdoerfer scores within similar external ear phenotypes indicates that external morphology alone cannot reliably characterize the underlying temporal bone anatomy. This distinction is relevant to the multidisciplinary evaluation of patients with microtia, in whom CT-based assessment can provide anatomical information relevant to surgical planning and hearing rehabilitation [17,18,29,30]. Accordingly, external ear classification may provide useful phenotypic context but should not substitute for individualized CT-based anatomical assessment when imaging is clinically indicated. However, the present study did not evaluate surgical or hearing outcomes or whether CT findings altered treatment decisions; therefore, the clinical implications of the observed phenotype–CT relationships should be interpreted within these limits.

Several limitations should be acknowledged. First, the retrospective, single-center design and relatively small sample size limit the generalizability of the findings. The small numbers within several phenotypic subgroups, particularly Marx Grade II (*n* = 6), Grade IV (*n* = 5), and the Nagata concha and small-concha subtypes (*n* = 3 each), further limit the precision of between-group comparisons and reduce the statistical power. Second, because analyses were performed at the ear level, the inclusion of one bilateral case may introduce limited within-subject non-independence. Although the patient-level sensitivity analysis addressed this issue by excluding one ear from the bilateral case, the inverse Spearman association was attenuated and the Jonckheere–Terpstra trend no longer reached statistical significance, reinforcing the need for cautious interpretation of the primary ear-level findings. Third, the use of a composite radiological score may not fully capture structure-specific developmental variability. In addition, the study did not evaluate hearing outcomes, surgical outcomes, or the influence of CT findings on treatment decisions. Finally, the requirement for both available preoperative external ear photographs and temporal bone CT examinations may have introduced selection bias and may limit the representativeness of the study cohort. Larger multicenter studies with more balanced phenotypic subgroups and linkage of anatomical findings to clinical outcomes are needed to determine the reproducibility and clinical relevance of the observed associations.

Overall, the present findings suggest a possible relationship between greater external auricular severity and less favorable CT-based temporal bone anatomy, but this relationship was not uniform across successive Marx grades and was attenuated in the patient-level sensitivity analysis. The substantial overlap in Jahrsdoerfer scores within external ear categories further indicates that phenotypically similar ears may differ considerably in their underlying temporal bone anatomy. Marx and Nagata classifications characterize different aspects of the external ear phenotype and may therefore provide complementary clinical descriptions; however, neither classification consistently reflected the extent of CT-defined temporal bone malformation in this cohort. These findings should be regarded as preliminary and support the need for larger studies to clarify phenotype–CT relationships while reinforcing that external ear phenotype should not be used as a substitute for individualized CT-based anatomical assessment when imaging is clinically indicated.

## 5. Conclusions

The present findings suggest a possible association between greater external auricular severity and less favorable CT-based temporal bone anatomy in patients with microtia; however, this relationship was not uniform across successive Marx grades. Substantial overlap in Jahrsdoerfer scores was observed within external ear phenotypic categories, and the association was attenuated in the patient-level sensitivity analysis. Marx and Nagata classifications characterize different aspects of external ear phenotypes, but neither consistently reflected the extent of underlying temporal bone malformation in this cohort. These preliminary findings indicate that external ear phenotype may provide contextual anatomical information but cannot substitute for individualized CT-based assessment when imaging is clinically indicated. Larger studies are required to clarify the relationship between external ear phenotype and temporal bone anatomy and to determine its clinical relevance.

## Figures and Tables

**Table 1:** Demographic and clinical characteristics of patients with microtia.

Variable	Value
Patients, *n*	26
Ears, *n*	27
Age, median (IQR), years	14 (11–18)
Male patients, *n* (%)	19 (73.1%)
Female patients, *n* (%)	7 (26.9%)
Bilateral involvement, *n* (%)	1 (3.8%)
Complete external auditory canal atresia, *n* (%)	24 (88.9%)
Stenotic but present external auditory canal, *n* (%)	3 (11.1%)

Each affected ear was analyzed as a separate unit in ear-based analyses. External auditory canal findings are reported per affected ear (*n* = 27).

**Table 2:** Distribution of Jahrsdoerfer scores according to Marx microtia grade.

Marx Microtia Grade	Ears (*n*)	Jahrsdoerfer Score, Median (Q1–Q3)	Range
Grade II	6	6.0 (3.75–9.0)	1–10
Grade III	16	8.0 (2.75–9.0)	1–9
Grade IV	5	1.0 (1.0–2.0)	1–5

Lower Jahrsdoerfer scores indicate less favorable temporal bone anatomy.

**Table 3:** Association between Marx microtia grade and radiological severity category.

Marx Microtia Grade	Severe (<5)	Moderate (5–8)	Mild (>8)
Grade II	2	2	2
Grade III	5	4	7
Grade IV	4	1	0
Total	11	7	9

Data are presented as numbers of affected ears. Radiological severity categories were defined according to Jahrsdoerfer score as severe (<5), moderate (5–8), and mild (>8).

## Data Availability

All data supporting the findings of this study are available upon request.

## Abbreviations

The following abbreviations are used in this manuscript:

CT	Computed Tomography
